# Longitudinal Intra- and Inter-individual variation in T-cell subsets of HIV-infected and uninfected men participating in the LA Multi-Center AIDS Cohort Study

**DOI:** 10.1097/MD.0000000000017525

**Published:** 2019-10-11

**Authors:** Najib Aziz, Beth D. Jamieson, Joshua J. Quint, Otoniel Martinez-Maza, Marianne Chow, Roger Detels

**Affiliations:** a Department of Epidemiology, UCLA Fielding School of Public Health; b Department of Medicine; c Departments of Obstetrics & Gynecology and Microbiology Immunology and Molecular Genetics, David Geffen School of Medicine at UCLA, Los Angeles, CA.

**Keywords:** biological variation, CD3, CD4, CD8, lymphocyte subsets, T-cell

## Abstract

To assess the intra-individual and inter-individuals biological variation and the effect of aging on lymphocyte T-cells subsets.

We assessed lymphocyte phenotypes (CD3^+^, CD4^+^, and CD8^+^ T-cells) in 89 HIV-1-infected and 88 uninfected white non-Hispanic men every 6 months, to examine the biological variation for those measurements, and the average change in lymphocyte phenotype over 34 years.

The markers showed significant intra-individuality in HIV-infected and uninfected individuals with index of individuality of <1.4. No mean changes were seen over the 34 years, with the exception of percentage CD4^+^T-cells in HIV-uninfected individuals.

In the **pre-HAART** era, HIV-infected individuals experienced an increase in mean absolute CD3^+^ T-cell numbers (11.21 cells/μL, *P* = 0.02) and absolute CD8^+^ T-cell numbers (34.57 cell/μl, *P* < .001), and in the percentage of CD8^+^ T-cells (1.45%, *P* < .001) per year and a significant decrease in mean absolute CD4^+^ T-cell numbers (23.68 cells/μl, *P* < .001) and in the percentage of CD4^+^ T-cells (1.49%, *P* < .001) per year.

In the **post-HAART** era, no changes in mean levels were observed in absolute CD3^+^ T-cell count (*P* = .15) or percentage (*P* = .99). Significant decreases were seen in mean count (8.56 cells/μl, *P* < .001) and percentage (0.59%, *P* < .001) of CD8^+^ T-cells, and increases in mean absolute count (10.72 cells/μl, *P* < .001) and percentage (0.47%, *P* < .001) of CD4^+^ T-cells.

With the exception of CD4 (%), no average changes per year were seen in lymphocyte phenotype of HIV-uninfected men. The results of coefficients of variation of intra and inter-individuals of this study can be useful for HIV-1 infection monitoring and in addition the observation could be a useful guide for intra- and inter-individual coefficient variations, and establishing quality goal studies of different blood biomarkers in healthy and other diseases.

## Abstract 2

Supplemental Digital Content is available in the text

## 1 Introduction

Analyte components of blood cells can fluctuate over the course of an individual's lifespan. Some of these variations are caused by predicable biological cycles or rhythms that all individuals share, while other sources of variation may be due to differences between individuals.

Numerous factors can contribute to variations in blood markers. Some of them are controllable (blood collection technique) whereas other characteristics such as race, sex, and age are not subject to manipulation. Biological factors are often the most important source of variation over time for certain analytes and marked changes can occur during the neonatal period, childhood, puberty, menopause, and aging. In addition, certain analytes have biological rhythms that can vary diurnally, monthly, or seasonally.^[1]^

The interpretation of a patient's test result using a laboratory's reference value is thus far the only valid tool available to clinicians when the biological variability of the population reference values and of the target population are comparable.^[2]^ Certain analytes display a high degree of inter-individual variation as a result of widely ranging homeostatic set points, even within a population that is considered “healthy.” This marked individuality makes it difficult to construct a single reference interval that represents a “healthy” range for all individuals since many individuals will present with values that are highly unique for them but still lie outside or inside population-based reference value ranges.^[3–10]^

Comparing a patient's test result with a value from a published reference population, it is possible to detect clinically relevant changes in blood lymphocyte subsets, which can be useful for prevention, diagnosis, prognosis, and treatment of various diseases. Repeated measurements of an analyte obtained by a longitudinal study of an individual may be preferable to use than a single measure in conjunction with population-based references. In the case of repeat measures, the patients have their own reference or baseline for each biomarker and changes between consecutive tests may be indicative of illness.^[2]^

Between 1979 and 1981, the appearance of opportunistic infections and Kaposi sarcoma in previously healthy gay men in the United States was documented as an acquired cellular immunodeficiency syndrome (AIDS) and the first human disease caused by an unknown agent with a significant loss of CD4^+^ T-cells.^[11–12]^

Cohort studies such as the MACS, WITS (Women and Infant Transmission Study), WIHS (Women's Interagency Health Study) and others, which investigated the natural history of human immunodeficiency type 1 (HIV-1) infection, discovered the utility of quantifying CD4^+^ T-cell numbers as a predictor of clinical risk in HIV-1 infected individuals, independent of treatment,^[13]^ and as a prognostic tool for HIV-1 disease progression.^[14–15]^ In addition to CD4^+^ T-cell depletion, lymphoid atrophy and immune activation, such as elevated expression of CD38 on CD8^+^ T-cells, are the other major characteristics of HIV-1 disease.^[16]^ In the early stages of HIV-1 infection, HIV RNA level has high prognostic value and in the later stages of disease, CD4^+^ T-cell count is a predictor of AIDS.^[14,17]^

Knowledge of the intra- and inter-individual variability are essential for the longitudinal assessment of blood biomarkers. Thus, the aim of our study was to assess the biological variation and the effect of aging on T-cells subsets (CD3^+^, CD4^+^ and CD8^+^ T-cell) over a period of 34 years. We accomplished this by examining the biological coefficient of variation of intra-individual (CV_I_) and inter-individuals (CV_G_) for immunophenotyped T-cell subsets in a population of HIV-1 infected and HIV-1 uninfected white non-Hispanic males. The biological variation and magnitude of changes in CD3^+^ T-cells, CD4^+^ T-cells, and CD8^+^ T-cells (percentages and absolute counts) were investigated in this study.

## 2 Material and methods

### 2.1 Study participants

We investigated routine lymphocyte phenotype in 2 groups (HIV-1 uninfected and HIV-1 infected individuals). The HIV-1 uninfected group consisted of 88 individuals with no diagnosis of major illness, such as cancer, hepatitis B or C infection, kidney disease or diabetes, all of whom were documented to be HIV-1 sero-negative at every study visit (6-month intervals) over the course of 34 years. The HIV-1 infected group was 89 individuals, of whom 2 participants did not receive any medication for HIV-1 until recent visits (1 of the participants started medication on February 2017), 19 received 1 to 3 different anti-viral medications, 30 received 4 to 6, 26 received 7 to 9, 9 received 10 to 12 and 3 received 13 to 15 in the period of 34 years.

These individuals were men who have sex with men (MSM) participating in the Los Angeles (LA) Multicenter AIDS Cohort Study (MACS) and self-report as white, non-Hispanic^[18]^ and for minimizing of inter-racial variability, other ethnicity were excluded. The age of the participants in the HIV-uninfected group at the start of the study (1984–1985) ranged from 22 to 49 years old with a mean of 33 and median of 32 years old. They were 51 to 81 years old at the last recorded visit through December 2017. The age of participants in the HIV-infected group at the start of the study (1984–1985) ranged from 22 to 56 years old with a mean of 31.5 and median of 31 years of age; they were 55 to 84 years old at the last recorded visit through December 2017. The average length of time between a participant's first and last visit was 34 years. The institutional review board for human studies at UCLA approved the protocols.

### 2.2 Blood collection and laboratory assays

After informed consent, blood samples were obtained every 6 months, between 8:00 am to 12:00 pm, from each individual into two of 4 ml (Becton Dickinson VACUTAINER Systems, New Jersey) tubes with EDTA anticoagulant for complete blood count (CBC) and flow cytometry analysis.

*Complete blood count (CBC)* assessments with 3-part differential and platelet count were performed with automated hematology analyzers by CLIA-certified clinical reference laboratories.^[19]^ The absolute count of a lymphocyte phenotype using white blood cell (WBC) count and lymphocyte percentage from the CBC report of each individual was calculated as follow: 
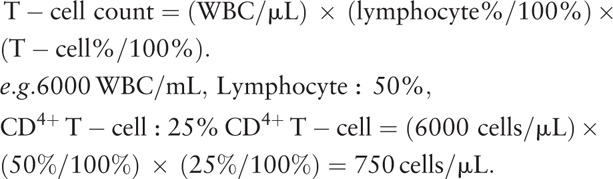


*Lymphocyte Immunophenotyping* performed by the Los Angeles MACS flow cytometry laboratory which participates in the Immunology Quality Assessment (IQA) program of NIAID/DIAIDS, using the Coulter EPICS Flow Cytometry System (April 1984 to April 1991), Becton Dickinson (BD) FACScan Flow Cytometry System (May 1991 to October 2004), and BD FACSCalibur Flow Cytometry System (October 2004 to present). Flow cytometry data were analyzed by utilizing Cellquest^R^ software (BDIS).

Throughout our study due to innovation in the field of laboratory instruments and automation technology, our laboratory used 3 different Flow cytometers. Each time the tests for accuracy, and precision were performed concurrently using the same samples by both instruments and the specifications set by manufacturer establishing the comparability overtime of the measurements.

### 2.3 Staining methods

EDTA Anticoagulated blood was collected by venipuncture and held at room temperature until staining, which was performed within 24 hours of collection. An ammonium chloride-lysed whole blood method (LW) and a Lyse no wash (LNW) method were performed. The detailed procedure can be found in published literature,^[20]^ briefly:

*Two-color Lyse wash staining*: (1984 to March 1996) 50 μl of undiluted whole blood was added to 12 × 75 mm tubes containing 20 μl of undiluted BD Simultest CD45PE/CD14 FITC CD3PE/CD4FITC, and CD3PE/CD8 FITC antibodies (BD BioScience), the tubes vortexed gently, and then incubated for 20 minutes at room temperature in the dark. After incubation, the cells were lysed twice by addition 1 ml of 1x freshly made ammonium chloride-lysing solution into^[20]^ the tubes and tubes were incubated for 5 and 3 minutes for the first and second washes respectively. At the end of each incubation, the tubes were centrifuged at 200g for 5 minutes and the supernatants removed without disturbing the cell pellets. After the final centrifugation, the pellet was re-suspended in 500 μl staining buffer (1 x PBS, 2% newborn calf serum, 1% Sodium azide).

*Lyse No Wash staining procedure*: (April 1996 to present time) 50 μl of undiluted whole blood was added to 12 × 75 mm tubes containing 20 μl of undiluted BD Tritest CD3FITC/CD4PE/CD45 PerCP and BD Tritest CD3FITC/CD8PE/CD45PerCP antibodies (BD BioScience), the tubes vortexed gently, and then incubated for 15 minutes at room temperature in the dark. After the incubation, 450 μl 1x BD FACSLyse lysing solution was added followed by another vortexing and 15 minutes incubation.

### 2.4 Statistical analysis

Descriptive statistics of the lymphocyte immunophenotyping markers that comprised the CD3^+^, CD4^+^, and CD8^+^ T-cells (absolute cell counts and percentage) were generated for mean and absolute ranges of intra- and inter-individuals. Linear trends were estimated using generalized estimating equations (GEE). We added a spline knot at visit 23 (1995–1996) to create a piece-wise regression function for calculating the average change. The spline function accommodated the comparison of the Pre-HAART and post-HAART period for HIV-1 infected individuals.

The nested analysis of variance was used for calculation of coefficients of variation of intra-individual (CV_I_) and inter-individuals (CV_G_). CV_I_ and CV_G_ were calculated according to the approach used by Harris and Boyd.^[21]^ Since the precision of the measurement tool was high, and the reliability of replicate measures was not of interest, we omitted measures of analytic variation from these calculations. Index of Individuality (II) is the simple ratio of the 2 biological components of variation: intra-individual to inter-individuals and is calculated using the formula CV_I_/CV_G_.^[1]^ The II, as defined by Harris^[22]^ assesses the usefulness of population-based reference values for interpretation of laboratory tests. If the II of a given analyte is greater than 1.4, then population-based intervals are useful; an II below 1.4 indicates decreased utility of population-based reference intervals. Analytes with an II less than 0.6 demonstrate (paradoxically) a high degree of individuality, making individual-based reference intervals more useful.

All analyses were performed using SAS version 9.4 (SAS Institute, Cary, NC). Graphs were created using SigmaPlot software version 14 (Jandel Scientific, San Rafael, CA 2018).

## 3 Results

### 3.1 Components of biological variation

The CV_I_, CV_G_, and index of individuality (II), along with the overall means (number and percentage) of T-cell lymphocyte phenotypes (CD3, CD4, CD8), for 1 (0–2 visits), 10 (0–20 visits), 20 (0–40 visits) and 34 (0–67 visits) years of follow-up for HIV-1 uninfected and infected individuals are presented in Table 1. As described in the methods, it is considered appropriate to use population-based reference ranges when the II of a given analyte is greater than 1.4. The indices of individuality in our study for the routine T-cell markers were less than 1.4 for all time intervals after year 1.

**Table 1:**
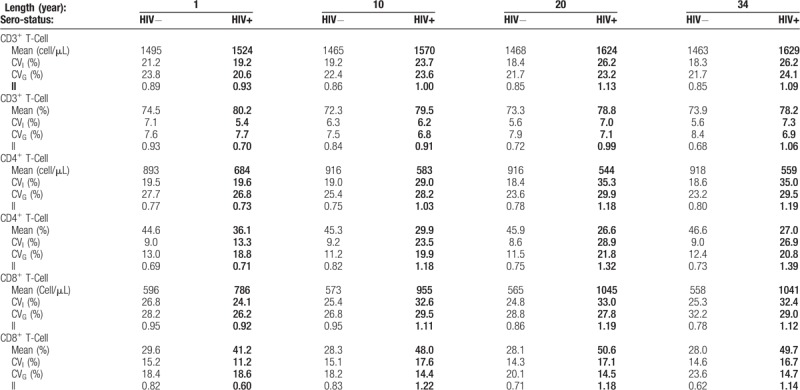
Mean values, intra-individual (CV_I_) and inter-individuals (CV_G_) variation, and index of individuality (II) for each T-cell parameter for 88 HIV-uninfected & 89 HIV-infected individuals at intervals from 1 to 34 years.

### 3.2 Mean and minimum-maximum T-cell values

**Figure 1:**
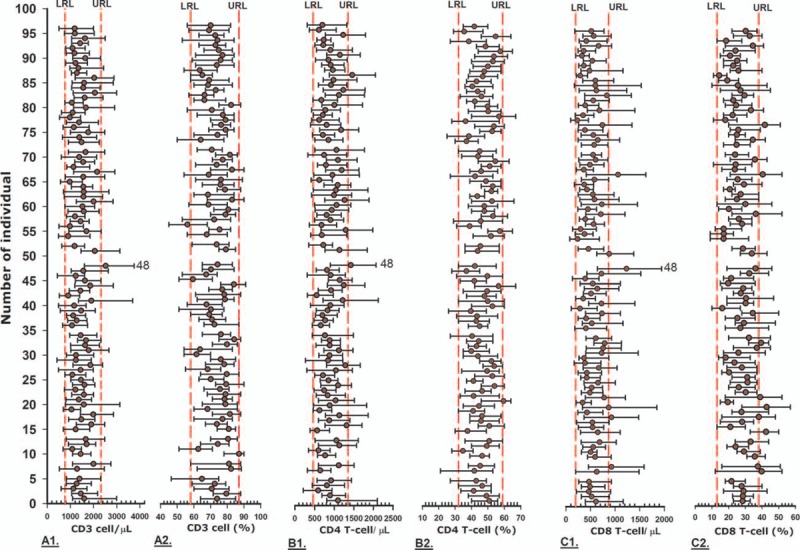
CD3^+^, CD4^+,^ and CD8^+^ T-cell of HIV-1 uninfected individual. Mean (filled circle) and absolute range (error bars) for each HIV-1 uninfected individual (n = 88) over 34 years follow-up, for absolute (A1, B1, and C1) count and percentage (A2, B2, and C2) of CD3^+^ T-cell, CD4^+^ T-cell, and CD8^+^ T-cell; each pair of vertical dashed lines represents the lower reference limit (LRL, left side) and upper reference limit (URL, right side), as described in the methods.

**Figure 2:**
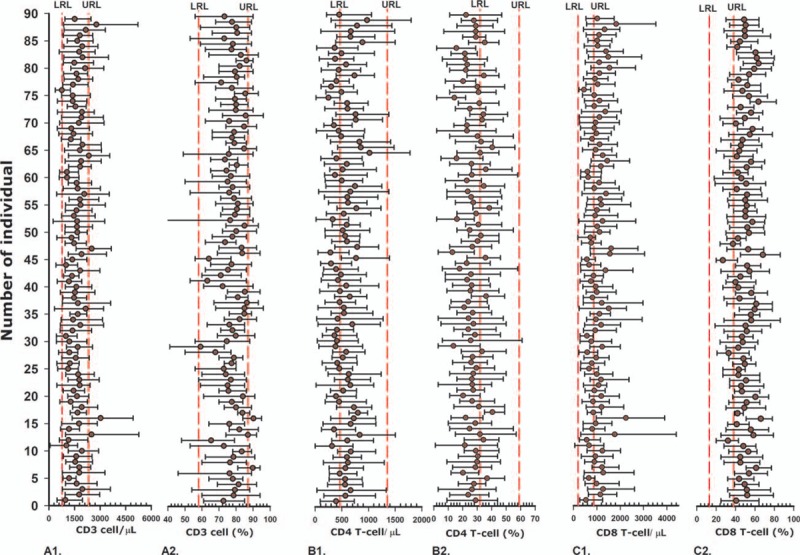
CD3^+^, CD4^+,^ and CD8^+^ T-cell of HIV-1 infected individual. Mean (filled circle) and absolute range (error bars) for each HIV-1infected individuals (n = 89) over 34 years follow-up, for absolute count (A1, B1,and C1) and percentage (A2, B2 and C2) of CD3^+^ T-cell, CD4^+^ T-cell, and CD8^+^ T-cell; each pair of vertical dashed lines represents the lower reference limit (LRL, left side) and upper reference limit (URL, right side), as described in the methods.

The means, absolute count and percentage ranges (minimum–maximum values) over all study visits for each HIV-1 uninfected individual (n = 88) and infected individual (n = 89) are shown graphically for CD3^+^ T-cells, CD4^+^ T-cells, and CD8^+^ T-cells in Figure 1 and Figure 2, respectively. Visual inspection of these figures shows that the mean values of the same analyte can differ greatly between individuals and this is seen in both the HIV-1 uninfected and infected groups.

The mean values of absolute counts and percentage of CD3^+^ T-cells for HIV-1 uninfected and infected individuals almost all lie within the reference limits (Fig. 1, 2 A1 and A2). Similarly, most mean values of absolute number and percentage of CD4^+^ T-cells and CD8^+^ T-cells for HIV-1 uninfected individuals lie within the reference interval. Looking at the data ranges, much of the data also fall within the reference intervals. However, there are definitely HIV-1 uninfected individuals with some data points that fall outside 1 or both of the upper and lower reference limits, for example absolute numbers of CD3+, CD4+, and CD8+ T-cells of participant of 48 (Fig. 1 A1, B1, C1).

28 (31%) out of 89 mean absolute counts of CD4^**+**^ T-cell data for HIV-1 infected individuals fell outside of the lower reference limit (LRL). Interestingly, we observed that 71 (79.8%) out of 89 mean percentages of CD4^+^ T-cell data fell outside the LRL (Fig. 2, B1 and B2). 67 (75%) out of 89 mean absolute counts of CD8^+^ T-cell data were outside the upper reference limit (URL) and for the percentage data, 85 mean percentages of CD8^+^ T-cell fell outside of the URL (Fig. 2, C1 and C2).

### 3.3 Magnitude of T-cell changes

In order to evaluate direction and magnitude of changes in each marker from baseline (0 visit) over the 34 years follow-up, generalized estimating equations in SAS software were used to estimate the average change per year in values and corresponding *P* values (Table 2). The longitudinal analysis over the course of 34 years, HIV-1 uninfected group showed a statistically significant increase *(0.1%/year)* only for percentage of CD4^+^ T-cells and no changes for other T-cell phenotypes in the HIV-1 uninfected individuals (Table 2).

**Table 2:**
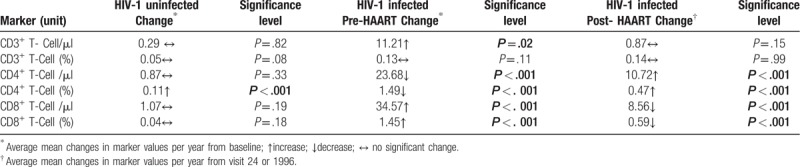
Average change per year in lymphocyte phenotype over 34 years follow-up of HIV-1 uninfected and infected men receiving HIV-1 highly active antiretroviral therapy (HAART).

The HIV-1 infected group in the pre-HAART era, from baseline (visit 0) to visit 23 (1996), a significant *decrease* was observed in HIV-1 infected individuals for both absolute count (23.7 cells/year) and percentage (1.5%/year) of CD4^+^ T-cells. Increases in values were seen for both absolute number (34.6 cells/year) and percentage (1.5%/year) of CD8^+^ T-cells during the same time period. A significant increase of 11.2 cells/year was seen for CD3^+^ T-cells, while no change was seen for CD3^+^ T-cell percentage per year (Table 2). In the post-HAART era, visit 24 to visit 67 (1996 -2018), a significant *increase* was observed for both absolute count (10.7 cells/year) and percentage (0.5%/year) of CD4^+^ T-cells. Significant decreases were seen for both absolute number (8.6 cells/year) and percentage (0.6%/year) of CD8^+^ T-cells while no **s**ignificant changes were seen for the CD3^+^ T-cell subset (Table 2).

## 4 Discussion

Flow cytometry has advanced from a limited research tool in the 1980s to a routine laboratory technique used today that, in addition to determining lymphocyte phenotype subsets, can be used to provide useful diagnostic and prognostic information about HIV-1 infection, leukemia, lymphoma, and other diseases. Additionally, serial or longitudinal testing of patients’ blood lymphocyte phenotype can assist physicians in detecting changes in markers over time and during the natural course of a disease aiding in treatment and preventative decisions.

For 34 years, we have longitudinally examined the mean values and biological variation of CD3^+^, CD4^+^, and CD8^+^ T-cells of lymphocytes circulating in the blood of HIV-1 uninfected and infected individuals in a relatively homogeneous cohort of men as they aged. We assessed the intra-individual coefficient of variation (CV_I_) and the inter-individual coefficient of variation (CV_G_) of absolute count and percentage of the lymphocyte phenotype parameters for 1, 10, 20, and 34 years of follow-up (Table 1).

The mean data of HIV-1 uninfected individuals during 1 year follow-up in our study for percentage and absolute counts of CD3^+^ T-cells (75%, 1495 cells/μl), CD4^+^ T-cells (45%, 893 cells/μl), and CD8^+^ T-cells (30%, 596 cells/μl) were consistent with the published studies of Valiathan et al. (78%, 1514 cells/μl for CD3^+^ T-cells, 47%, 921 cells/μl for CD4^+^cells, and 28%, 562 cells/μl for CD8^+^ T-cells),^[23]^ Tollerud et al (75%, 1582 cells/μl for CD3^+^ T-cells, 49%, 1036 cells/μl for CD4^+^T-cells, and 28%, 595 cell/μl for CD8^+^ T-cells),^[24]^ and Reichert et al (73% for CD3^+^ T-cell, 43% for CD4^+^T-cells, and 33% for CD8^+^ T-cells).^[25]^ Despite the testing only males, our data reiterated the technical reliability, and biological stability of the CD3^+^, CD4^+^, and CD8^+^ T-cells of lymphocytes circulating in the blood of the above published studies.

In 1994, Hughes et al evaluated the magnitude of the CV_I_ of CD4^+^ T-cell count in asymptomatic HIV-1 infected individuals (2 year follow-up) for 3 groups based on their absolute CD4 counts of 200, 500, and 800 cells/μl. The CV_I_ of the study were 35%, 25%, and 19%, respectively^[26]^ and the third group has some similarity with CV_I_ of CD4^+^ T-cells of HIV-1 infected individuals our study (19.6% and mean CD4^+^ T-cells of 684 cells/μl).

The mean and ranges of lymphocyte phenotype are graphically presented in Figure 1 and Figure 2 for HIV-1 uninfected and HIV-1 infected individuals, respectively. The mean absolute counts and percentage values of CD3^+^, CD4^+^, and CD8^+^ T-cells were relatively variable and yet, for the HIV-1 uninfected individuals, a majority of data fell within the conventional reference interval value and any illnesses or problems they might have had were not caught using the lymphocyte phenotype conventional reference values. In those circumstances, using the patient's own reference or baseline for the lymphocyte subsets may be a useful tool for follow-up.^[4]^

In HIV-1 infected individuals, the absolute counts and percentage data of CD3^+^ T-cells are varied and unusual but still fall within the reference interval value; this may be due to homeostatic compensation of mostly of CD8^+^ T-cell changes in HIV-1 infected individuals.

Using CD4^+^ or CD8^+^ T-cell percentage data (Supplementary Fig. 1C and E) along with the absolute counts of CD4^+^ or CD8^+^ T-cells (Supplementary Fig. 1 D and F) as a laboratory tool can add more prognostic value compared to using either absolute values or percentages alone. This is particularly evident when dealing with CD4+ T-cell counts of HIV-1 infected individuals (Supplementary Fig. 2 A and B).

The direction and magnitude of changes for each lymphocyte phenotype marker from baseline (0 visit) over the 34 years follow-up, reflecting, among other factors, the aging of the individuals was also studied for HIV-1 uninfected and infected individuals. With the exception of the percentage of CD4^+^ T-cell increase per year (*P* < .001), there were no significant average changes per year for absolute counts and percentage of CD3^+^ T-cells, CD8^+^ T-cells, and absolute count of CD4^+^ T-cells over 34 years for HIV-1 uninfected individuals (Table 2) (Supplementary Fig. 1).

HIV-1 infected individuals showed an interesting pattern of a deflection point (downwards) for percentage and absolute counts of CD4^+^ T-cells and inflection point (upwards) for percentage and absolute counts of CD8^+^ T-cells at time point of 12 years (1995–1996 or HAART era) of 34 years follow-up. The period between baseline to 12 years coincided with the pre-HAART era and the time of 1996 to 2017 was the post- HAART era of HIV-1 infected individuals.

CD4+ T-cells in HIV-1 infected individuals, the data show a significant average decrease of 23.68 absolute cell numbers and 1.49% per year from the baseline mean to 12 year follow-up (deflection point) and a subsequent and significant average increase of 10.72 cells and 0.47% from mean value of the 12-year time point to 34 years follow-up (post-HAART). The reverse was seen for CD8^+^ T-cells, with a significant average increase of 34.57 cells and 1.45% per year from the baseline mean to 12 year follow-up and a significant average decrease of 8.56 cells and 0.59% per year from mean value at the 12-year time point (inflection point) to 34 years follow-up (post-HAART) (Table 2 and supplementary Fig. 1).

**Figure 3:**
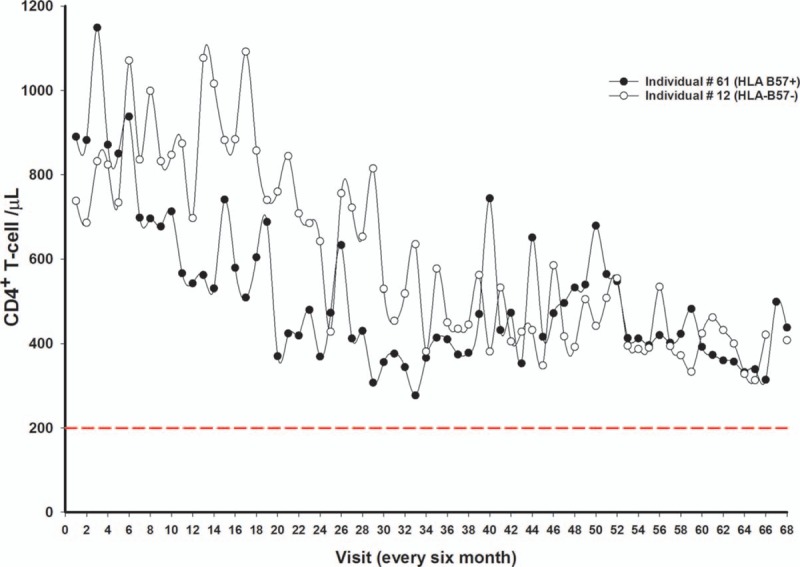
Absolute count changes of CD4^+^ T-cells. Filled circles symbolize HIV-1 infected individual # 61 (HLA-B57^+^) with a baseline CD4^+^ T-cell count of 890 cell/μl who started HIV-1 medication on February 2017. Open circles symbolize HIV-1 infected individual # 12 with a baseline CD4^+^ T-cell count of 738 cell/μl followed for total of 68 visit (34 years). The dashed red line represents the CD4^+^ T-cell limit for an AIDS diagnosis.

Two HIV-1 infected individuals, # 61 and # 12, 1 of whom was HLA-B57+ (Individual # 61) had CD4^+^ T-cell counts of 890 and 738 cells at baseline, respectively. Both were long-term non-progressors (LTNP's) maintaining a favorable course of asymptomatic infection with high CD4^+^T-cells and low viral load.^[27–28]^ They did not receive any anti-HIV-1 medication in the course of the 34 years follow-up (individual # 61 has taken medication since February 2017), and did not follow the same pattern (deflection or inflection) as the rest of the HIV-1 infected group, showing instead a constant slow decrease in percentage and absolute counts of CD4^+^ T-cells (Fig. 3).

## 5 Conclusion

The lymphocyte phenotypes (CD3, CD4, CD8) in HIV-1 uninfected individuals show high individuality but with a majority of values falling within the reference range and for those individuals, knowledge of baseline levels will be a helpful tool for follow-up. Thirty-four years of follow-up demonstrate that the mean values of CV_I_ are higher, relative to CV_G,_ values in HIV-1 infected individuals as compared to HIV-1 uninfected individuals. CD4^+^ T-cell percentage value, along with the absolute counts of CD4^+^ T-cells, adds more prognostic value compared to using either absolute values or percentages alone when dealing with CD4^+^ T-cell counts of HIV-1 infected individuals. With the exception of CD4 (%), no average changes per year were seen in lymphocyte phenotype subsets of HIV-1 uninfected men and the data were stable over the course of 34 years. These results can be useful for informing prognosis or outcome of therapy. In addition the observations could be a useful guide for intra- and inter-individual coefficient variations, and establishing quality goal studies of different blood biomarkers in healthy and other diseases.

## Acknowledgments

We thank the men who participate in the MACS, who make this and many other studies possible. We also thank Timothy Ryner for manuscript help, Patricia Hultin, Lance E. Hultin for technical support, Adrian Cornejo and Rey Soto for their contribution to data analysis.

## Author contributions

**Conceptualization:** Najib Aziz, Beth D. Jamieson, Otoniel Martinez-Maza, Roger Detels.

**Data curation:** Najib Aziz, Marianne Chow.

**Formal analysis:** Joshua J. Quint.

**Investigation:** Roger Detels.

**Methodology:** Najib Aziz, Beth D. Jamieson, Marianne Chow.

**Software:** Najib Aziz, Joshua J. Quint.

**Visualization:** Najib Aziz.

**Writing – original draft:** Najib Aziz.

**Writing – review & editing:** Najib Aziz, Beth D. Jamieson, Joshua J. Quint, Otoniel Martinez-Maza, Marianne Chow, Roger Detels.

Najib Aziz orcid: 0000-0002-8907-8730.

## Supplementary Material

Supplemental Digital Content
