# Supplementary material for: Longitudinal Intra- and Inter-individual variation in T-cell subsets of HIV-infected and uninfected men participating in the LA Multi-Center AIDS Cohort Study
Source: Medicine (Baltimore). 2019 Oct 11;98(41):e17525. doi: 10.1097/MD.0000000000017525 (PMC6799419; doi:10.1097/MD.0000000000017525)

**Supplementary Fig. 1. Percentage and absolute count changes of lymphocyte phenotype CD3^+^, CD4^+^, and C8^+^ T-cells**. Linear plots display changes in the means every six months for thirty-four years for HIV-1 uninfected individuals (filled circle) and HIV-1 infected individuals (open circle) in lymphocyte subsets of CD3^+^ T-cell % (A) and CD3^+^ T-cell count (B); CD4^+^ T-cell % (C) and CD4^+^ T-cell count (D); and CD8^+^ T-cell % (E) and CD8^+^ T-cell count (F). ↑ represents the start point (1995-1996) of highly active antiretroviral therapy (HAART).


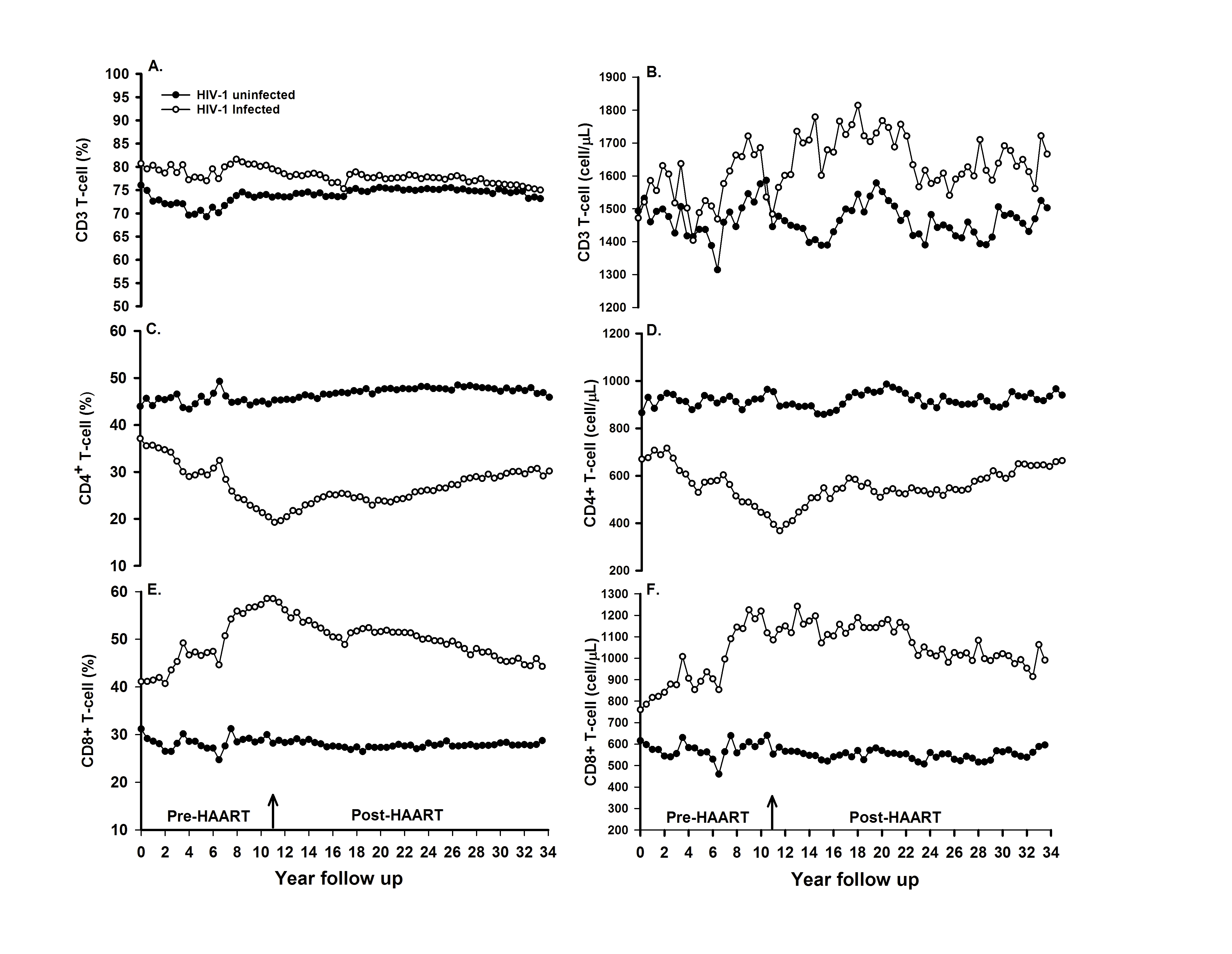


**Supplementary Fig. 2. Percentage and absolute count changes of CD4^+^ T-cells.** Open circles in plot A represent the percentage of CD4^+^ T-cells and in plot B the absolute counts of CD4^+^ T-cells for each visit of 89 HIV-1 infected individuals over the course of 34 years.


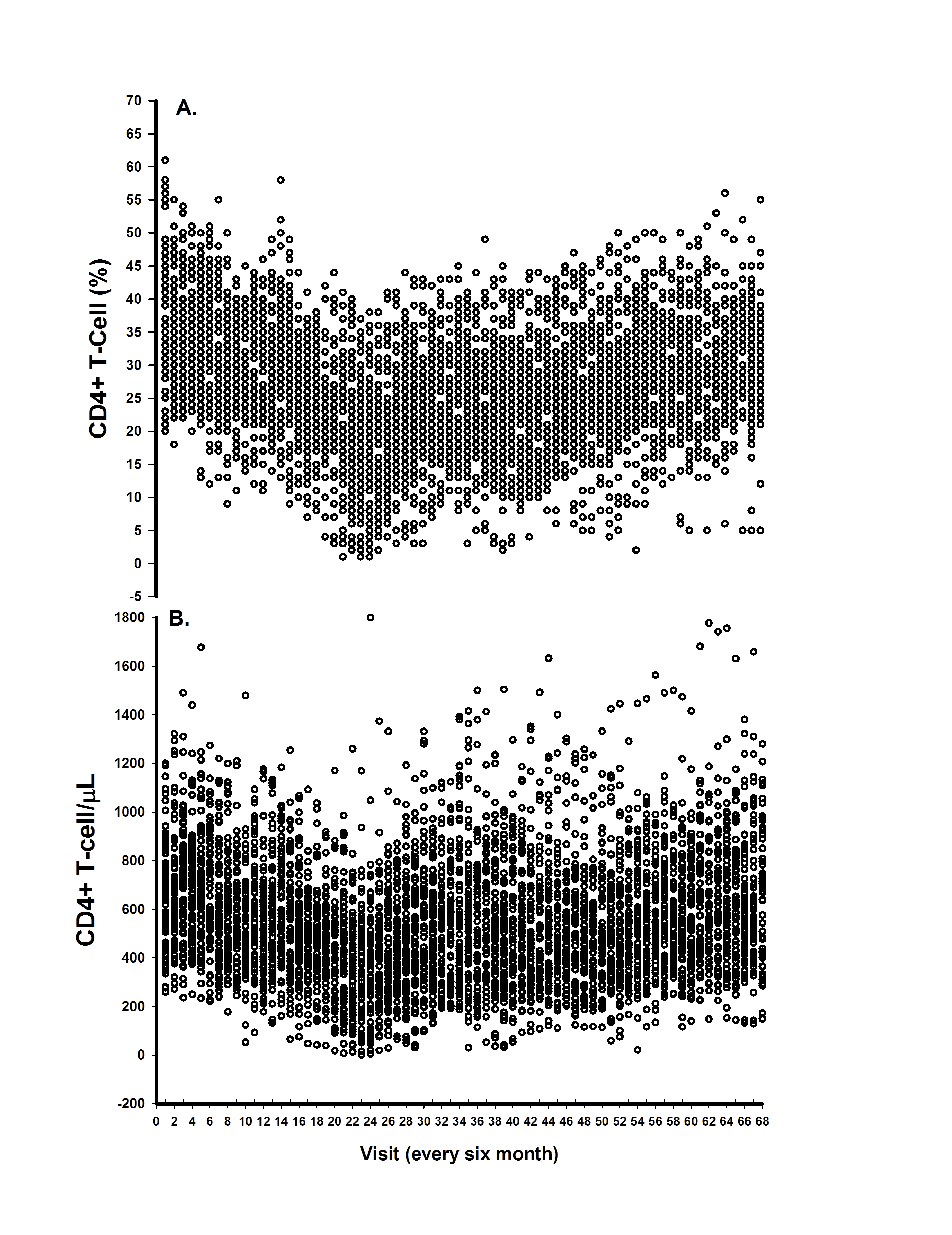

Supplement: Supplemental Digital Content [file medi-98-e17525-s001.doc]
